# Control of the Verticillium Wilt on Tomato Plants by Means of Olive Leaf Extracts Loaded on Chitosan Nanoparticles

**DOI:** 10.3390/microorganisms10010136

**Published:** 2022-01-10

**Authors:** Elisabetta Mazzotta, Rita Muzzalupo, Adriana Chiappetta, Innocenzo Muzzalupo

**Affiliations:** 1 Centro di Ricerca Olivicoltura, Frutticoltura, Agrumicoltura, Consiglio per la Ricerca in Agricoltura e l’Analisi dell’Economia Agraria (CREA-OFA), C.da Li Rocchi-Vermicelli, 87036 Rende, CS, Italy; mazzotta-elisabetta@libero.it; 2 Dipartimento di Farmacia, Scienze della Salute e della Nutrizione, Universitá della Calabria (DFSSN-UNICAL), Ed. Polifunzionale, 87036 Arcavacata di Rende, CS, Italy; rita.muzzalupo@unical.it; 3 Dipartimento di Biologia, Ecologia e Scienza della Terra, Università della Calabria, Cubo 6B, 87036 Arcavacata di Rende, CS, Italy

**Keywords:** antifungal activity, in vivo experiments, plant disease, sustainable strategies, polyphenols activity

## Abstract

In this research, a new ecofriendly and sustainable fungicide agent, with the ability to control Verticillium wilt, was developed. To this purpose, a green extract of olive leaf (OLE) was prepared by ultrasound-assisted extraction (UAE) and characterized in terms of polyphenol content and antioxidant activity. Then, OLE was loaded in chitosan nanoparticles (CTNPs) to combine the antifungal activity of CTNPs and phenolic compounds to obtain an important synergic effect. Nanoparticles were synthetized using the ionic gelation technique and characterized in terms of sizes, polydispersity index, Z-potential, encapsulation efficiency, and release profile. Qualitative and quantitative analyses of OLE were performed by the HPLC method. OLE-loaded CTNPs exhibited good physicochemical properties, such as a small size and positive surface charge that significantly contributed to a high antifungal efficacy against *Verticillum dahliae*. Therefore, their antifungal activity was evaluated in vitro, using the minimal inhibition concentration (MIC) assay in a concentration range between 0.071 and 1.41 mg/mL. Free OLE, blank CTNPs, and OLE-loaded CTNPs possessed MIC values of 0.35, 0.71, and 0.14 mg/mL, respectively. These results suggest an important synergic effect when OLE was loaded in CTNPs. Thereafter, we tested the two higher concentrations on tomato plants inoculated with *V. dahliae*, where no fungal growth was observed in the in vitro experiment, 0.71 and 1.41 mg/mL. Interestingly, OLE-loaded CTNPs at the higher concentration used, diminished the symptoms of Verticillium wilt in tomato plants inoculated with *V. dahliae* and significantly enhanced plant growth. This research offers promising results and opens the possibility to use OLE-loaded CTNPs as safe fungicides in the control strategies of Verticillium wilt at open field.

## 1. Introduction

Verticillium wilt is a devastating soil-borne fungal disease in various plant species that represents a serious threat to agricultural productivity. The Verticillium wilt is caused by colonization of plant xylem vessels of a pathogenic fungus, *Verticillium dahliae*. As a result, the vascular system of the plants is altered, the supply of water and nutrients is limited, and chlorosis or defoliation as well as plant death occurs [1]. Verticillium affects more than 200 economically important plants worldwide, including alfalfa, cotton, cucurbits, eggplant, mint, potato, sunflower, strawberry, olive, and tomato with billions of dollars lost [2].

Moreover, tomato is a model crop to study plant-pathogen interactions. Every year, tomato is inflicted with several pathogens, including Verticillium that limits its production. Therefore, the control of *V. dahliae* pathogen is essential [3].

Olive oil production is one of the main agro-industries in Mediterranean countries. In addition, the production of olive oil is increasing, as well as the amount of olive oil by-products, such as wastewater and leaves, which can cause environmental problems. The olive leaves extracts may aid in the treatment of a broad range of diseases and the bioactive properties are attributable primarily to the phenolic content [4].

Different ecofriendly strategies have been developed to control plant diseases. Particular attention has been addressed to sustainable practices, to avoid the use of chemical pesticides, such as the use of resistant varieties, crop rotations, and biocontrol agents [5].

In the last years, particular advantages in the control of plant diseases caused by a fungal pathogen is the employment of engineered nanomaterials. Nano-sized particles, thanks to their different properties with respect to bulk materials, such as high surface area, small sizes, and high reactivity, offer a wide range of promising use for the delivery of fertilizers, herbicides, pesticides, and fungicides. In agriculture, nanoparticles have shown promise for their small sizes that enhance the penetration into the plant cell, which can, in turn, increase the agrochemicals uptake. The encapsulation of agrochemicals in nanoparticles also reduces nutrient losses, improves solubility and nutrient uptake, and reduces the rate of application of traditional fertilizers and pesticides [6].

Particularly interesting in this field are chitosan nanoparticles (CTNPs). Chitosan is a natural, biocompatible, non-toxic polymer with intrinsic antimicrobial activity. A wide range of applications has been proposed for CTNPs. CTNPs can be used as an encapsulating agent to produce slow-release fertilizer [7] and as a material with the ability to alleviate abiotic stress in plants, by means of salinity control and the alleviation of drought stress [8]. Chitosan is widely used itself as a plant growth promoter, thanks to its positive nature, resulting in an increased affinity to the plant cell membrane and, thus, an enhanced reactivity [9]. Moreover, CTNPs have found large application as a soil conditioner to complex toxic metals and their removal in polluted soil [10] and as a bioflocculant in wastewater treatments [11].

The direct use of chitosan as an antifungal agent has been limited by its water insolubility. However, the manipulation of this material in the nano-scale range makes it an attractive candidate for agriculture use [12]. Numerous studies have demonstrated the antimicrobial activity of CTNPs against fungi [13,14,15,16]. The antifungal effectiveness of CTNPs was determined to be dependent on the particle size and Zeta potential. Zeta potential refers to the surface charge of nanoparticles and is an important parameter influencing the stability and biological activity [17]. A smaller particle size and high positive surface charge are commonly associated with a better inhibition effect of fungal growth. This could be explained due to the high surface area of nanosized particle, which is available to more effectively interact with the fungus with respect to the free form of chitosan. Moreover, the positive charge enhances the interaction with a negative-charged fungi cell membrane, altering membrane permeability and causing cell death [18].

Chitosan based nanomaterials used in combination with other antimicrobial agents showed an enhanced and synergic antimicrobial effect [19]. Natural compounds have gained attention in the agricultural practices as potential antimicrobial agents of pathogenic fungi and bacteria. Various studies have demonstrated that phenolic compounds can significantly contribute to the biological control of pathogenic microorganisms and substitute or support the activity of common synthetic fungicides [20]. Byproducts of the agro- and food industry are excellent sources of phenolic compounds, which exert antimicrobial activities. For example, olive leaves are the major waste product in the olive oil production, containing a considerable amount of bioactive natural compounds. Among these, phenolic compounds have a large amount of biological activities. Additionally, in the last years, an increasing interest has been addressed to use these compounds in agri-food, cosmetic, and pharmaceutical industries. Oleuropein is the main active compound in olive leaves with interesting pharmacological and health-promoting properties, which is an important antimicrobial activity against bacteria and fungi [21]. Recently, interest has been addressed in the valorization of these residues to achieve extracts enriched in phenolic compounds to produce high value-added products. Furthermore, the development of a sustainable and cost-effective extraction technique of phenolic compounds from olive leaf is crucial to extend their application in sustainable agriculture. Several extraction methods have been employed [22,23], and particularly interesting is ultrasound-assisted extraction (UAE), a promising, inexpensive, and rapid technique to obtain a high value-added compound using ecofriendly solvents [24].

Therefore, in this work, an investigation has been conducted to assess the antifungal activity of polyphenol compounds extracted with a green technique from olive leaves. Our purpose was to encapsulate the obtained extract into CTNPs to combine the potentiality of chitosan polymer with phenolic molecules and to evaluate them as biofungicides against Verticillium wilt, which is induced by the pathogen *V. dahliae* using tomato as a model plant.

## 2. Materials and Methods

### 2.1. Chemicals

Chitosan, 2,2-diphenyl-1-picrylhydrazyl (DPPH), 2,2-azinobis-(3-ethylbenzothiazoline-6-sulfonate) (ABTS), sodium triphosphate (TPP), Folin Ciocalteau reagent, tyrosol, coumaric acid, ferulic acid, were purchased from Sigma-Aldrich (Milan, Italy), luteolin, verbascoside, luteolin-7-O-glucoside, rutin, oleuropein, luteolin-4-O-glucoside, ligstroside, luteolin all purchased from Extrasynthése (Genay, Francia).

B’cuzz Hydro A + B nutrient solution (A: N-P-K 5-0-5; Nitrogen (N) 4.85%, Phosphorus pentoxide (P_2_O_5_) 0.15%, Potassium Oxide (K_2_O) 4.73%, Sodium Oxide (Na_2_O) 0.19%, Calcium Oxide (CaO) 3.79%, Magnesium Oxide (MgO) 1.32%, Sulfur Trioxide (SO_3_) 0.11%, Iron (Fe) 0.040%, Boron (B) 0.001%; B: N-P-K 0-4-6; Nitrogen (N) 0.68%, Phosphorus pentoxide (P_2_O_5_) 4.07%, Potassium Oxide (K_2_ O) 5.71%, Sodium Oxide (Na_2_O) 0.20%, Calcium Oxide (CaO) 0.25%, Magnesium Oxide (MgO) 0.71%, Sulfur Trioxide (SO_3_) 0.94%, Manganese (Mn) 0.029%, Copper (Cu) 0.001%, Zinc (Zn) 0.039%, Molybdenum (Mo) 0.001%, Boron (B) 0.012%) ATAMI, Rosmalen, Netherlands.

All of the reagents for microbiology were used and provided by Oxoid Limited (Basingstoke, UK). All of the solvents for the high-performance liquid chromatography were used and provided by VWR International Srl (Milan, Italy). Moreover, all of the chemicals were used at 99% purity.

### 2.2. Preparation and Characterization of Olive Leaf Extract

Polyphenol extraction from olive leaves of cv Coratina was carried out, according to the protocol reported by Difonzo et al. [25] with some modifications. Olive leaf extract (OLE) was prepared by the UAE technique using a water:ethanol solution (30:70 (*v*/*v*) as solvent, with other parameters set on the best conditions that affect the yield of the polyphenolic content previously studied [26]. Specifically, the extraction was performed at 25 °C for 20 min using an olive leaf mass-to-solvent volume ratio 1:5 (*w*/*v*). The liquid extract was centrifuged at 10,000× *g* for 10 min, filtered (0.45 µm), dried with a rotavapor, lyophilized, and stored at 4 °C in the dark, prior to the experiments.

High performance liquid chromatography (HPLC) analysis was performed for the analytical qualification and quantification of phenolic compounds in OLE, according to the IOOC method [27]. The extracts (5 mg) were dissolved in 80% methanol (1 mL) and filtered with 0.45 μm filters before the analysis by HPLC (UV–Vis). The HPLC apparatus (1100 Series, Agilent, Milan, Italy) with a spectrophotometric UV detector at 280 nm and an integrator using a column Inertsil ODS-2 (5 μm, 15 cm Å~4.6 mm i.d.) was equipped with a Spherisorb S5 ODS-2 (5 μm, 1 cm Å~4.6 mm i.d., Sigma-Aldrich, srl, Milano, Italy) precolumn, at a temperature of 40 °C. The elution was carried out in gradient mode using a binary solvent mixture composed of water acidified with 0.2% phosphoric acid (solvent A) and methanol/acetonitrile 50/50 (solvent B). A linear gradient was run from 96% (A) and 4% (B) to 50% (A) and 50% (B) for a duration of 40 min. Then, it changed to 40% (A) and 60% (B) for 5 min, and for the duration of 15 min, it changed to 0% (A) and 100% (B), after re-equilibration for 12 min to an initial composition. The mobile phase flow rate was 1 mL/min and the injection volume of each sample was 20 μL. All of the phenolic compounds were identified by comparing their retention times with those of standards (luteolin, hydroxytyrosol, tyrosol, coumaric acid, ferulic acid, verbascoside, luteolin-7-O-glucoside, rutin, oleuropein, oleuropein aglycon, luteolin-4-O-glucoside, ligstroside, luteolin).

The Folin-Ciocalteu method was used for the determination of total polyphenolic content. One milliliter of the extract was mixed with 0.5 mL Folin-Ciocalteu and left in the dark for 5 min. Therefore, 3 mL of 20% Na_2_CO_3_ and 5.5 mL of distilled water were added and left for 20 min to react. The absorbance of the blue intense color that developed was measured at 765 nm using a UV-Vis spectrophotometer. The calibration curve was plotted using a standard oleuropein and the data are expressed as milligrams oleuropein equivalents (OE) per grams of extract. The correlation coefficients of the calibration curve ranged between 0.9994 and 0.9997.

The antioxidant activity of the OLE was evaluated using DPPH and ABTS assays. For the DPPH assay, the method reported by Mazzotta et al. was used [28]. Briefly, 1.5 mL of the extract at different OLE concentrations were incubated with 1.5 mL of ethanol DPPH solution (0.25 mM) at room temperature in the dark. After 30 min, absorbance measurements were taken at 517 nm with UV-Vis spectrophotometer. The DPPH radical scavenging activity was calculated according to the following equation:

(1) $$Scavenging\;activity\;\left(\%\right)=\frac{\left(A{}^{\circ}-A^{1}\right)}{A{}^{\circ}}\times 100$$

where A° is the absorbance of control (blank, ethanol) and A^1^ is the absorbance in the presence of the extract. Each experiment was carried out in triplicate and the results were expressed as the mean ± SD.

The antioxidant activity of the extract was also estimated by measuring its capacity to scavenge free radical ABTS• [29]. A stock solution of 7mM ABTS was mixed in equal quantities with potassium persulfate (2.45 mM) and stirred in the dark for 12 h to generate ABTS• free radicals. Prior to use, the solution was diluted with ethanol to obtain an absorbance of 0.708 ± 0.001. Then, 0.5 mL of the samples were added to 2.5 mL of the ABTS•+ solution. After 7 min, the absorbance was evaluated at 734 nm using a UV spectrophotometer.

Finally, the ABTS scavenging activity was calculated, using the following equation:

(2) $$\%\;inhibition=\left(\frac{A_{0}-A_{S}}{A_{0}}\right)\times 100$$

where A_s_ is the absorbance of the sample at 734 nm and A_0_ is the control. All of the tests were realized in triplicate and the results were expressed as the mean ± SD.

### 2.3. Preparation and Characterization of OLE-Loaded Chitosan Nanoparticles

CTNPs were prepared using the ionic gelation technique reported by Muzzalupo et al. [30]. The chitosan low molecular weight was solubilized in HCl solution 0.04% (*v*/*v*) achieving a concentration of 1 mg/mL. Briefly, 1 mL of the extract (10 mg/mL) was added, and the pH of the resulting solution was adjusted to 5.5 with NaOH 1 M. Then, 1 mL of aqueous solution of sodium triphosphate (TPP) was dropwise added and the resulting solution was stirred at room temperature for 30 min. CTNPs formation was spontaneous thanks to the ability of positive-charged chitosan in establishing electrostatic interactions with the negative-charged crosslinking agent.

Of note, to investigate the CTNPs uptake by plants, CTNPs were prepared in the same manner by adding 0.5 mL of [9-(2-carboxyphenyl)-6-diethylamino-3-xanthenylidene]-diethylammonium chloride (Rhodamine-B) ethanol solution (2 mg/mL) into a chitosan solution.

The size and Zeta potential of the CTNPs were determined, at 25.0 °C, by dynamic and electrophoretic light scattering (DLS) analysis using a 90 Plus Particle Size Analyzer (Brookhaven Instruments Corporation, New York, NY, USA) and Zeta-sizer ZS (Malvern Instruments Ltd., Malvern, UK), respectively. The OLE-loaded CTNPs morphology and size were also analyzed using transmission electron microscopy (TEM) (Jeol 1400 Plus electron microscope, JEOL Ltd., Milano, Italy) after the treatment with a 2% phosphotungstic acid solution. The extract encapsulation efficiency was indirectly determined by measuring the free un-trapped extract. CTNPs were filtered using the syringe filters with a porosity equal to 0.22 µm (Millipore, Roma, Italy) to separate the non-encapsulated extract from the CTNPs, and then the amount of free extract was analyzed using UV spectrophotometry at 280 nm. The entrapment efficiency was calculated according to the following formula:

(3) $$EE\%=\frac{\mathrm{Initial}\;\mathrm{extract}\;\mathrm{amount}-\mathrm{free}\;\mathrm{extract}\;\mathrm{amount}}{\mathrm{Initial}\;\mathrm{extract}\;\mathrm{amount}}\times 100$$

### 2.4. In Vitro Release of OLE from CTNPs

The OLE release from CTNPs was carried out in a sink condition in a dialysis bag at room temperature. Briefly, 2 mL of CTNPs was dialyzed in 50 mL in a phosphate buffer (PBS) 0.1 M pH 5.9 (90 mL of NaH_2_PO_4_ H_2_O 0.2 M was mixed with 10 mL NaH_2_PO_4_ 7 H_2_O 0.2 M, and then diluted up to 200 mL). In addition, at set time intervals, 2 mL of the medium was taken and replaced with the same volume of a fresh buffer. The solution was analyzed using the UV–Vis spectrophotometer at 280 nm. The amount of OLE release was determined according to a calibration curve, prepared using OLE solutions of known concentration in the appropriate range. All of the experimental procedures were repeated three times, and the results agreed within the 4% standard deviation limit.

### 2.5. CTNPs Uptake Experiments

CTNPs uptake experiments were carried out according to Luo et al. [31] with some modification. Columbia ecotype *Arabidopsis* plants were used for the experiment. Seeds were sterilized and grown as reported in [32]. Briefly, the seeds were washed in absolute ethanol for 2 min, then in 1.75% of hypochlorite solution (NaClO) for 12 min, and finally, three times in sterile distilled water for 5 min.

The seeds were sown on Petri dishes and germinated on a 0.7% agar medium enriched with 1% sucrose and micro- and macro-nutrients (1/2 Murashige and Skoog basal salt, Sigma-Aldrich, Florence, Italy). Plated seeds were left at 4 °C for 48 h to synchronhyze the germination in a growth chamber at 21 °C, under 16/8 h light/dark photoperiod (150 µmol/m^2^s) and 60% of relative humidity.

After germination, *Arabidopsis thaliana* roots of 5-day-old seedlings were immersed for 30 min in Rhodamine-B-labelled CTNPs (0.3 mg/mL) solution. Thereafter, the roots were washed with distilled water and the uptake and translocation of the nanoparticles were analyzed through a high-resolution laser confocal microscope (Confocal Microscope Leica TCS SP8). Rhodamine-B was imaged using the excitation method at 543 nm and fluorescence detection at >560 nm.

### 2.6. In Vitro Isolation of V. dahliae (AACC0021)

To evaluate the antifungal activity of OLE-loaded CTNPs, the *V. dahliae* (AACC0021) strain was used. The isolation and identification of the strain were described in a previous study [33]. This strain was sub-cultured on potato dextrose broth (PDB) and incubated in darkness at 24 °C for 21 days. Briefly, 20 µL of the suspension was taken to prepare the slides using a Malassez cell. The samples were observed under an optical microscope (DMRB Leica Microsystems, Milan, Italy) at 400× magnification, equipped with a digital camera (DFC490 Leica Microsystems) and conidiospores were quantified. Thereafter, the samples were divided into 1.5 mL aliquots and stored at −80 °C in 25% of glycerol.

### 2.7. In Vitro Antifungal Susceptibility Testing: Determination of the Minimal Inhibitory Concentration

The minimal inhibitory concentration (MIC) of fungal growth was evaluated according to the national committee for clinical laboratory standards (NCCLS) using the broth dilution method [5,34]. Conidiospores of fungal strains were diluted in PDB at a concentration of 10^6^ conidiospores/mL at 26 °C prior to use. Free OLE and OLE-loaded CTNPs were serially diluted in PDB, in order to obtain OLE concentrations equal to 1.41 (a), 0.71 (b), 0.355 (c), 0.142 (d), and 0.071 mg/mL (e). The same dilutions and conditions were also performed for blank CTNPs. Then, 1 mL of each sample was incubated in a tube with 1 mL of *V. dahliae* conidiospore suspension at a concentration of 10^6^ conidiospores/mL for 7 days at 25 °C. The MIC was measured as the highest dilution, in which no detectable growth of *V. dahliae* was observed.

### 2.8. In Vivo Plant Growth Conditions

Tomato (*Lycopersicon esculentum* L.) cv Chico III plants were chosen to test the antifungal efficacy of OLE-loaded CTNPs against *V. dahliae.* Prior to planting, seeds were surface sterilized by immersion in a solution of 3.5% sodium hypochlorite for 20 min [35]. A drop of Tween 20 was added, and then the seeds were rinsed three times in distilled water. Thereafter, the seeds were transferred to a germination chamber containing agriperlite sterilized by two cycles of autoclave at 121 °C and 1 atm for 40 min. Ten-day-old seedlings were transplanted into pots of 1 L, containing 1 kg of sterilized mixture of peat moss and sand (1:1 *v*/*v*). Plants were grown in Percival cabinets with a cycle of 16 h light (2.5 × 10^3^ µmol photons m^−2^ s^−1^) at 26 °C and 8 h dark at 22 °C. The application of the fertilizer was conducted weekly with B’cuzz Hydro A + B nutrient solution (ATAMI, Rosmalen, The Netherlands).

### 2.9. Plant Inoculation

Thirty tomato plants with three true leaves (above 25 days) were removed from the pots, and the roots were washed in distilled water to remove the soil. Therefore, the roots were dipped for 3 min in 0.5% of agarose gel containing 1 × 10^6^ spore/mL of *V. dahliae* (AACC0021) [36]. The plants were replanted in the pots and maintained as previously described. Ten tomato plants at the same developmental stage were used as healthy control and their roots were dipped in 0.5% of agarose gel (Sigma-Aldrich, Milano, Italy) without conidial suspension. The CTNPs treatment was performed after the appearance of symptoms on leaves at 10 days of inoculation. Three different groups of 10 plants each were treated as follows: (i) 10 mL/plant of distilled water, (ii) 10 mL/plant of OLE-loaded CTNPs at a concentration of 1.41 mg/mL, and (iii) 10 mL/plant of OLE-loaded CTNPs at a concentration of 0.71 mg/mL. All of the treatments were applied in the soil. Each plant was subsequently monitored 7 and 15 days after the CTNPs treatment. Additionally, the experiment was performed in triplicate.

A disease assessment on individual plants was carried out through the determination of wilt severity from four different expert observers. Wilt severity was rated using the scale adopted by Shittu et al. [36] with a score between 0–5 as follows: 0, plant healthy; 0.5, premature loss of both cotyledons; 1.0, yellowing and flaccidity of the first leaf; 2.0, lower 40% of leaves affected; 3, lower 60% of leaves affected; 4, lower 80% of leaves affected; 5, plant dead.

### 2.10. Identification of Fungal DNA in Infected Plant Tissue

DNA extraction was carried out using leaves of the first and second stem node. Fresh leaf tissue was homogenized in 600 µL of a CTAB buffer (100 mM Tris HCl pH 8.0, 1.4 M NaCl, 20 mM EDTA pH 8.0, 2% hexadecyltrimethylammonium bromide) using pre-chilled mortar and pestle. The samples were transferred in 2 mL eppendorf tubes and incubated at 65 °C for 30 min. Then, 600 µL of chloroform:iso-amyl alcohol (24:1) was added, mixed gently by vortex for 15 min at 40 rpm, and then centrifuged for 5 min a 12,000 rpm. The upper aqueous phase containing DNA was transferred into a clean tube, and 600 µL of cold isopropanol was added and incubated at −20 °C overnight to precipitate the DNA. The contents were mixed three to four times by inverting the tubes gently, and then centrifuged at 12,000 rpm for 10 min. The supernatant was decanted and the pellet was washed with 600 µL ice cold 70% of ethanol. Then, the supernatant was discarded, and the pellet was air dried at room temperature. The pellet was dissolved in a TE buffer and the DNA was quantified using Nanodrop ND-100 (Thermo Fisher Scientific, Waltham, MA, USA) spectrophotometer. The PCR assay was carried out using a Veriti^TM^ (Applied Biosystem, Waltham, MA, USA) thermal cycler for the specific identification of *V. dahliae*. The PCR primers targeted against *V. dahliae* (AACC0021) were VDS1: Forward 5′CACATTCAGTTCAGGAGACG3′ (Sigma-Aldrich, Florence, Italy) and VDS2: Reverse 5′CCGAAATACTCCAGTAGAAG3′ (Sigma-Aldrich, Florence, Italy) used at the final concentration of 0.2 µM [37]. PCR amplification was carried out in a final volume of 25 µL containing 12.5 µL of buffer, 0.5 µL of primers (10 µM), 2 µL of DNA, 0.4 µL of KAPA3G Plant PCR Kit (KAPA Biosystems, Inc., Boston, MA, USA), and 9.1 µL of distilled water. The amplification was performed using 35 reaction cycles consisting of a 30 s denaturation step at 95 °C, 30 s annealing steps at 63 °C, and 1 min elongation step at 72 °C [37]. Thereafter, 10 µL aliquots of the final product were electrophoretically analyzed on a 1% agarose gel stained with 12 µL of Sybr Safe DNA gel stain and then images of the gels were captured under UV light using the U:Genius imager (Syngene, Cambridge, United Kingdom). 

### 2.11. Statistical Analysis

Statistical analysis was performed using the one-way analysis of variance (ANOVA) and Bonferroni corrected *p*-value for the multiple comparison test. All of the results represent the % of at least three individual experiments and are expressed as the mean ± SD of three independent experiments.

## 3. Results

The extract was characterized in terms of polyphenols content and antioxidant activity to predict its potentiality as natural fungicide. Table 1 shows the quantification and identification of phenolic compounds by chemical analysis and revealed that oleuropein is the main component of the extract.

Total polyphenolic content, determined by the Folin-Ciocolteau method, was 693.45 ± 0.98 mg/g OE of dry olive leaf weight, indicating a phenolic-rich extract similar to the one obtained using the traditional extraction technique with an organic solvent [30].

**Figure 1:**
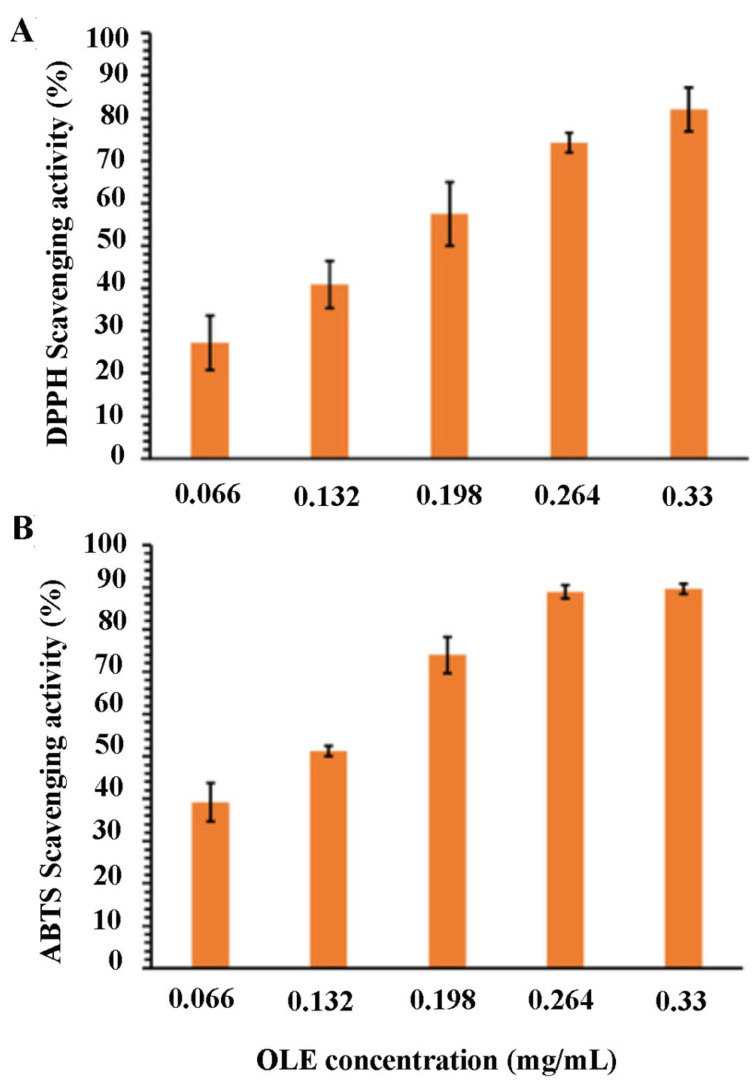
Antioxidant activity of OLE against DPPH• (**A**) and ABTS• (**B**) expressed as percentage of radical scavenging activity versus extract concentration. The results represent the mean ± SD of three independent experiments.

**Figure 2:**
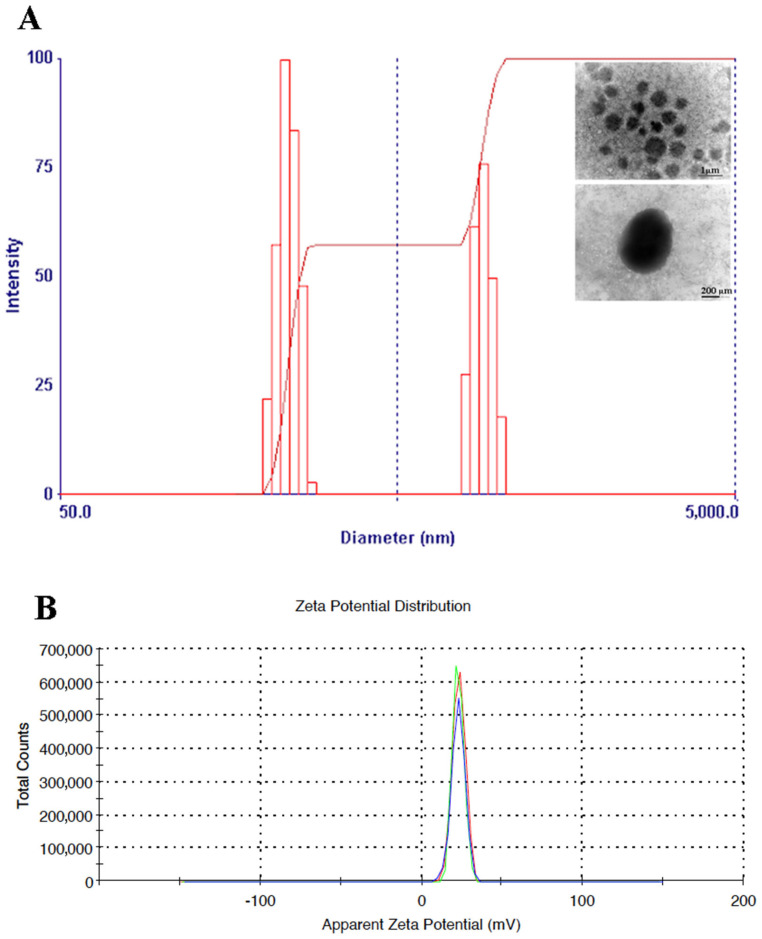
Physicochemical characterization of OLE-loaded CTNPs: (**A**) Particle size distribution by the DLS analysis and TEM micrographs; (**B**) Zeta potential distribution.

The results of the OLE antioxidant activity are shown in Figure 1A,B, demonstrating a strong antioxidant effect against DPPH• and ABTS• radicals. The size of OLE-loaded CTNPs was evaluated by the DLS analysis. The results showed that the obtained nanodevices had an average particle size of 331.26 ± 26.80 nm and a good uniformity of particle size distribution with a PDI of 0.231 ± 0.01 (Figure 2A). Furthermore, the Z-potential value of the obtained CTNPs was +21.1 ± 1.50 mV, indicating a good colloidal stability (Figure 2B).

The successful loading of OLE into CTNPs was confirmed by the UV–Vis analysis that recorded an EE% equal to 58.47 ± 6.36%, indicating a good OLE encapsulation efficiency.

**Figure 3:**
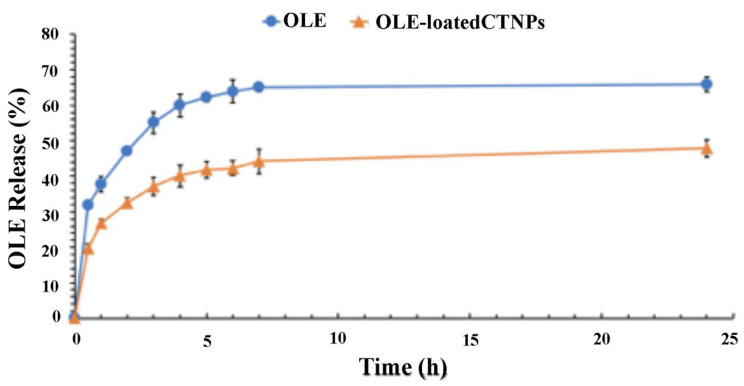
In vitro OLE release in phosphate buffer pH 5.9 at room temperature from OLE-loaded CTNPs (▲), and OLE solution (•). In all of the cases, each value represents the mean ± SD of three independent experiments.

As shown in Figure 3, the OLE release was slowed down by its encapsulation in CTNPs. In this way, a low release was recorded after 24 h both for free OLE and OLE-loaded CTNPs equal to 65.82% and 47.82%, respectively. Potentially, this behavior could be related to the low solubility of OLE lipophilic molecules in PBS that led to a slow diffusion. These results are in good agreement with a previous study, where a similar release trend was observed [30].

To verify the absorption and translocation of CTNPs in plant tissues, Rhodamine-B was encapsulated in CTNPs, and the resulting particles were used to investigate the distribution behavior in *Arabidopsis* root tissues.

**Figure 4:**
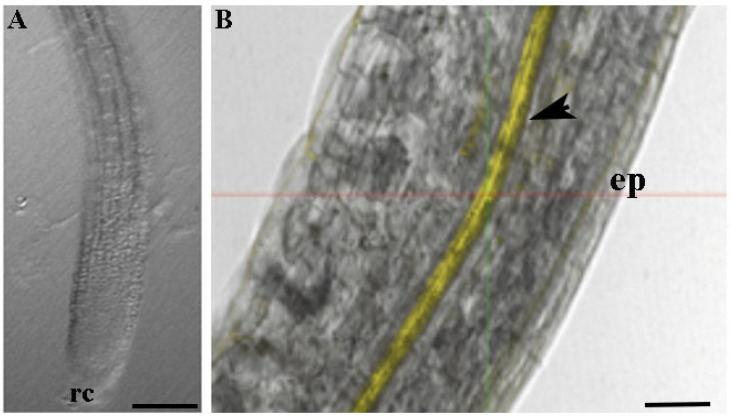
Confocal laser scanning microscopy images (**A**) non-treated and (**B**) treated with Rhodamine-B-labelled CTNPs. (**B**) Yellow signal is localized in the xylem of *Arabidopsis* root (arrowhead). ep: Epidermis; rc: Root cap. Scale bars (**A**) 150 μm; (**B**) 70 μm.

Figure 4 clearly shows the localization of nanoparticles in root plants. A uniform and thicker layer of yellow fluorescent signal was recorded, indicating that CTNPs, after loading at the level of absorbing region of the root, are translocated through the xylem tissues.

**Figure 5:**
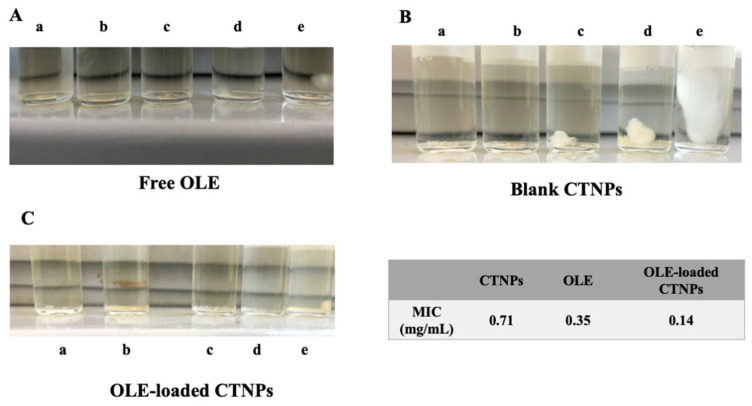
Minimal inhibition concentration (MIC) of OLE when free (**A**) and loaded in CTNPs (**B**); MIC of blank CTNPs was also evaluated at the same dilutions (**C**); a: 1.41 mg/mL; b: 0.71 mg/mL; c: 0.355 mg/mL; d: 0.142 mg/mL; e: 0.071 mg/mL.

The antifungal activity of OLE-loaded CTNPs was investigated in vitro by the determination of minimal inhibition concentration (MIC), using the broth dilution method [5,34]. The results are presented in Figure 5 and suggested a higher antimicrobial activity against *V. dahliae* of OLE-loaded CTNPs compared to free OLE and blank CTNPs. Indeed, OLE, CTNPs, and OLE-loaded CTNPs possessed MIC values of 0.35, 0.71, and 0.14 mg/mL, respectively (Figure 5).

Thereafter, pot experiments in a controlled environment chamber were carried out to verify the in vivo antifungal efficacy of the OLE-loaded CTNPs on tomato plants infected by *V. dahliae*. Plants inoculated with *V. dahliae* fungal strains after 10 days showed typical symptoms of *Verticillum* wilt, such as flaccidity, yellowing, stunting, as well as leaf wilting and chlorosis. The treatments with OLE-loaded CTNPs at concentrations of 1.41 and 0.71 mg/mL were carried out and data for disease severity after 7 and 15 days were recorded.

Preliminarily, to verify the infection of *V. dahliae* in inoculated and both non-symptomatic and symptomatic plants, we used conventional PCR with primers specific for fungus. A strong amplification signal was detected in inoculated plants (positive CTR), while a weak or no amplification signal was observed in plants inoculated with *V. dahliae* and treated with OLE-loaded CTNPs. No amplification was detected in non-inoculated plants (negative CTR) (Figure S1). The infection, evaluated by PCR methods on the total of plants, was detected in 70% of positive CTR, and in 60% of plants that tested positive to *V. dahliae* and treated with OLE-loaded CTNPs at a concentration of 0.71 mg/mL. On the contrary, the percentage of plants that tested positive to *V. dahliae* and treated with OLE-loaded CTNPs at a concentration of 1.41 mg/mL was significantly (*p* < 0.05) decreased to 45%.

**Figure 6:**
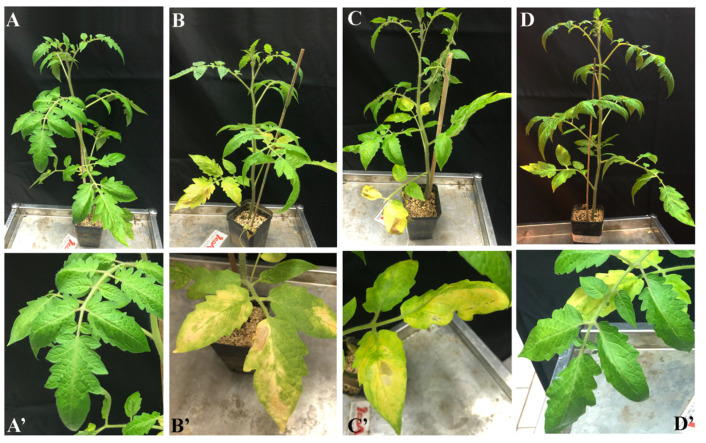
Tomato plants inoculated with *V. dahliae* for 10 days ((**B**,**B’**) Positive CTR) and treated with OLE-loaded CTNPs for 7 days at (**C**,**C’**): 0.71 mg/mL, (**D**,**D’**): 1.41 mg/mL, (**A**,**A’**) non-inoculated tomato plants (negative CTR).

Plants inoculated with *V. dahliae* (Figure 6B) for 10 days showed severe chlorotic mottling on individual leaves, followed by wilt and leaf drop with a disease score equal to 1.42 ± 0.50. No significant symptoms of recovery were observed after the treatment with OLE-loaded CTNPs at a concentration of 0.71 mg/mL for 7 days, with a disease score equal to 1.42 ± 0.32 (Figure 6C). In contrast, tomato plants infected with *V. dahliae*, and treated with OLE-loaded CTNPs at a concentration of 1.41 mg/mL for 7 days, showed an incidence of the disease as significantly reduced (*p* < 0.05), with a score of 1.08 ± 0.19 (Figure 6D).

**Figure 7:**
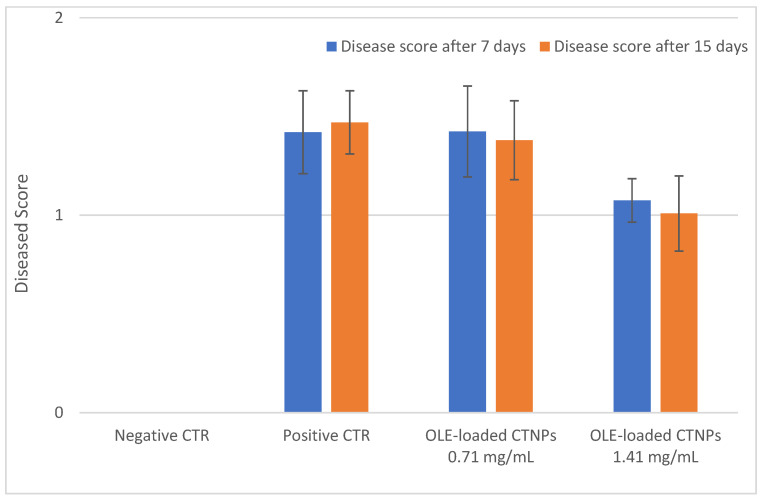
Symptoms of *V. dahliae* infections evaluated in seedlings of tomato at the three-leaf stage. Plants were scored at 7 (blue bars) and 15 (orange bars) days post-inoculation with the fungus. The results summarize the data for 10 plants per time point for each interaction.

The antifungal efficacy of OLE-loaded CTNPs on *V. dahliae* infected tomato plants was also evaluated 15 days after the treatment and the obtained results were similar to those observed at 7 days (Figure 7).

**Figure 8:**
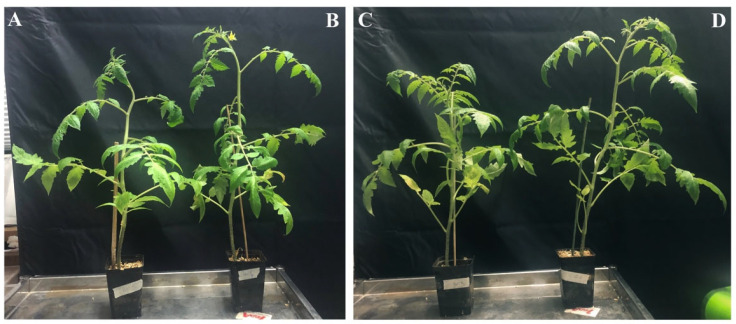
In vivo effect of OLE-loaded CTNPs on the growth state of tomato plants. (**A**) Plants inoculated with *V. dahliae* for 10 days (positive CTR); (**B**) non-inoculated plants (negative CTR). Plants inoculated with *V. dahliae* for 10 days and treated with OLE-loaded CTNPs; (**C**) at a concentration of 0.71 mg/mL and (**D**) at a concentration of 1.41 mg/mL.

Furthermore, the growth of tomato plants inoculated with *V. dahliae* was significantly affected by the OLE-loaded CTNPs treatment (Figure 8). Fifteen days after the treatment, the negative CTR plants showed more vigor than the *V. dahliae* inoculated plants (positive CTR) (Figure 8A). Similar results were obtained with the plant treated with lower CTNPs concentration (Figure 8B). In contrast, the infected plants treated with OLE-loaded CTNPs at a concentration of 1.41 mg/mL (Figure 8D) presented a higher stem length and leaf number compared to both the treated OLE-loaded CTNPs at a concentration of 0.71 mg/mL (Figure 8C) and the positive control (Figure 8A).

## 4. Discussion

In the present study, CTNPs loaded with OLE were proposed as new antifungal agents against *V. dahliae*. Verticillum wilt is a fungal disease that occurs on olive trees worldwide and represents one of the most severe olive problems. If uncontrolled, it can cause significant yield reduction. Consequently, an increased interest has been addressed to develop new sustainable practices based on the use of bio-friendly fertilizers and pesticides as a substitute to agrochemicals. Agriculture by-products are an important source of natural compounds with different biological activities. In addition, their transformation in high-value products could represent a potential eco-sustainable strategy. In this work, we exploit olive leaves as an inexpensive and renewable source of compounds with antifungal activity. OLE was produced using a green and environmentally friendly extraction method to reduce the generation of hazardous product and the negative environmental impact. OLE was characterized by chemical analysis and the obtained results are in line with the previously published data for *Olea europaea* leaves, where oleuropein was identified as the major phenol compound extract [25]. Moreover, the total polyphenolic content comparable to the content obtained with the organic solvent clearly confirmed that the proposed extraction technique represents a new, green, and inexpensive procedure, that has the ability to successfully recover active compounds from a natural source.

Considering that the antioxidant activity of phenolic compounds is widely demonstrated and the high phenolic content of our extract, its antioxidant potential was also investigated. The scavenging ability of DPPH• and ABTS• radicals resulted as dose-dependent, reaching DPPH and ABTS inhibition radicals’ percentage of 82.10% and 89.62%, respectively. Furthermore, OLE showed a different sensitivity to in vitro antioxidant tests. Considering the 50% of inhibition of free radicals as a function of the extract concentration, the IC_50_ of OLE were 0.114 and 0.168 mg/mL against the DPPH• and ABTS• radical, respectively. The lower IC_50_ against the ABTS• radicals highlight a better antioxidant activity, implying that OLE showed a stronger ability to scavenge ABTS• radicals. In fact, at a concentration of 0.198 mg/mL, the extract was shown as the ABTS scavenging activity of 73.93%. On the contrary, at the same concentration, the scavenging activity of DPPH• radicals resulted as only 57.49%. Then, the OLE extract was encapsulated in chitosan nanoparticles to produce stable and protective vesicles for agricultural applications. Recently, nanotechnology has generated relevant interest in the development of agricultural products, since nanoparticles are excellent tools to reduce both synthetic agrochemicals in agriculture practice and environmental impact, and to enhance the uptake of nutrients from the soil [6]. Furthermore, chitosan shows an intrinsic antifungal activity [7]. Therefore, the use of this polymer in combination with polyphenol compounds could represent a successful strategy to cope with fungal plant diseases.

Nanoparticles were prepared by an ionotropic gelification, a simple and rapid method based on the spontaneous electrostatic interaction between the cationic group of chitosan and anionic group of TPP without the use of organic solvent or surfactants. Nanoparticles were characterized regarding their physicochemical properties and their in vitro and in vivo antifungal effects. The particle size and surface charge are important parameters towards the development of suitable nanodevices, since they influence the in vivo distribution. These physicochemical properties are also a crucial factor affecting the antimicrobial activity and different studies demonstrated a better antimicrobial activity of small and positive-charged nanoparticles [38,39].

The results from the DLS analysis performed in this study, revealed a small hydrodynamic diameter of OLE-loaded CTNPs, representing an advantageous factor to develop tools with improved antifungal efficacy. Typically, the small particle size was related to a larger surface area. Therefore, more active sites are available for the cytotoxic interaction with fungus membrane. In addition, positive values of Z-potential, similar to those recorded for OLE-loaded CTNPs, are related with a higher ability to establish an electrostatic interaction with biological membranes and to improve cellular uptake and thereby, promote the antimicrobial activity [40,41].

In fact, the antifungal activity of CTNPs is due to the electrostatic interaction between the glucosamine amino group of chitosan with the negative-charge membrane of fungus [42,43], altering membrane permeability and resulting in cell death.

Consequently, particles that are developed in this research hold physical characteristics and structural features suitable for the development of an effective fungicide nanodelivery system. Moreover, it is important to evaluate the nanoparticle ability to translocate in plant tissue and to release antifungal agents to the target site. The obtained data from the performed experiments indicate that CTNPs can cross the cellular layer of the endoderm and reach the xylem. Moreover, a delayed release of OLE was recorded allowing for the sustained release in time and thus, avoiding an early OLE release before reaching the target disease. Therefore, OLE-loaded CTNPs were considerably stable before reaching the fungus site and may effectively control fungal disease by destroying and inhibiting the fungus after OLE release.

Fungicidal activity of blank CTNPs is widely reported [7] and our results confirmed this intrinsic and weak antifungal activity. Instead, the OLE extract showed a strong inhibition of fungal growth and this activity further increased when the extract was loaded in CTNPs. At a concentration of 0.14 mg/mL, a significant fungal growth was observed for CTNPs, while a weak growth was detected when OLE was used alone. On the contrary, no fungal growth occurred in OLE-loaded CTNPs. The best antifungal efficiency observed for OLE-loaded CTNPs could be due to a synergic effect of chitosan and phenolic compounds [19].

In vivo experiments were also performed to evaluate the OLE-loaded CTNPs antifungal activity, addressed to manage fungal wilt disease in agriculture practices. Disease control was significantly affected by the OLE-loaded CTNPs treatment and a clear positive correlation between the treatment concentration and disease index was observed. The plant response to Verticillium wilt was dependent on the concentration of OLE-loaded CTNPs used. For the plant treated with the lower OLE-loaded CTNPs (0.71 mg/mL), the incidence and severity of the disease were not reduced. Conversely, the increase in concentrations of OLE-loaded CTNPs led to a significant disease reduction and improvement of plant health. The treatment with OLE-loaded CTNPs at a concentration of 1.41 mg/mL strongly inhibited the growth of *V. dahliae* in tomato plants. Indeed, the OLE-loaded CTNPs treated plants appeared clear and healthy and showed a bright green color compared to the infected seedlings, indicating the possibility of their use to control phytopathogens and reduce the disease severity.

The observed fungicidal activity of OLE-loaded CTNPs may be attributed to several reasons, such as the positive nature and small sizes of nanoparticles. As reported in the literature, polycationic particles interact with the negative-charged membrane of fungi leading to cell lysis and disruption of membrane integrity. In addition, the particles size significantly affects the antifungal efficacy of NPs: Smaller particles have a larger surface area, which is available for interaction with the fungal membrane. Therefore, they are associated with a better uptake into microbial cells and more antimicrobial effect than the larger particles [17,44,45]. Another reason could be related to the ability of chitosan to activate plant immune response and enhance the activity of antioxidant and defense enzymes [46]. Chitosan biopolymer is indeed a strong elicitor of plant defense mechanisms, leading to the elevated antifungal activity in potted plants [47].

In addition to the antifungal activity, we also found a significant increase in growth parameters of tomato plants treated with OLE-loaded CTNPs at a concentration of 1.41 mg/mL. This effect may be due to the role of chitosan as a growth plant promoter, since it influences several plant physiological processes, such as nutrient uptake, cell division, cell elongation, enzymatic activation, and synthesis of protein [48]. Our results provide the evidence that OLE-loaded CTNPs have an important control activity of Verticillum wilt and represent an effective antifungal solution for the infected plants. Therefore, the proposed system could be successfully used as a versatile platform for fungicide and phytotherapeutic molecules in plant disease treatment.

## 5. Conclusions

In summary, a phenolic-rich extract with a strong antioxidant and antifungal efficacy was obtained in this research using a simple, inexpensive, and environment-friendly extraction technique. The antifungal efficacy of OLE was enhanced by loading it in CTNPs due to the important synergic effect of chitosan and phenolic compounds. The obtained results indicated that OLE-loaded CTNPs had a strong in vitro and in vivo antifungal activity against *V. dahliae*. The efficacy of OLE-loaded CTNPs to control Verticillium symptoms in tomato plant infected with *V. dahliae* resulted as dependent on the concentration used. In fact, only OLE-loaded CTNPs at a concentration of 1.41 mg/mL showed the effectiveness against the disease by *V. dahliae* and played an active role in disease reduction and enhancement of growth parameters. Therefore, OLE-loaded CTNPs at the higher concentration can be used as antifungal agents of choice against Verticillium wilt. Furthermore, they have a remarkable potential for further evaluation in field conditions as a safe alternative to chemical fungicides.

## Figures and Tables

**Table 1:** Relative quantities (%) of identified phenolic compounds in olive leaves extracts. Values are expressed as mg/g extract of oleuropein equivalents.

Phenolic Compounds	Retention Time (min)	%	Concentration (mg/g DW)
Hydroxytyrosol	7.66	4.64%	32.16 ± 0.33
Tyrosol	10.68	0.04%	0.28 ± 0.03
n.d.	12.12	2.54%	17.62 ± 0.22
Coumaric acid	14.28	0.24%	1.68 ± 0.11
Ferulic acid	15.65	0.24%	1.68 ± 0.09
n.d.	15.89	0.16%	1.12 ± 0.07
Verbascoside	16.12	3.83%	26.56 ± 0.24
Luteolin-7-glucoside	16.64	7.18%	49.77 ± 0.41
Rutin	17.11	1.09%	7.55 ± 0.19
Oleuropein	18.42	74.68%	517.85 ± 0.84
Oleuropein aglycon	19.24	0.08%	0.56 ± 0.01
n.d.	19.89	0.16%	1.12 ± 0.04
Luteolin-4-glucoside	20.21	2.18%	15.10 ± 0.18
Ligstroside	21.21	2.50%	17.34 ± 0.14
Luteolin	22.17	0.44%	3.08 ± 0.02
Total	-	100%	693.45 ± 0.94

The results represent the mean of three independent experiments. n.d.: Not detected.

## Data Availability

The data presented in this study are available on request from the corresponding author.

## Supplementary Materials

The following supporting information can be downloaded at: https://www.mdpi.com/article/10.3390/microorganisms10010136/s1. Figure S1: PCR amplification of *Verticillium dahliae* in tomato leaves.
