# Supplementary material for: Control of the Verticillium Wilt on Tomato Plants by Means of Olive Leaf Extracts Loaded on Chitosan Nanoparticles
Source: Microorganisms. 2022 Jan 10;10(1):136. doi: 10.3390/microorganisms10010136 (PMC8781408; doi:10.3390/microorganisms10010136)
Supplement: Supplementary file 1 [file microorganisms-10-00136-s001.zip › microorganisms-1515582-supplementary.pdf]

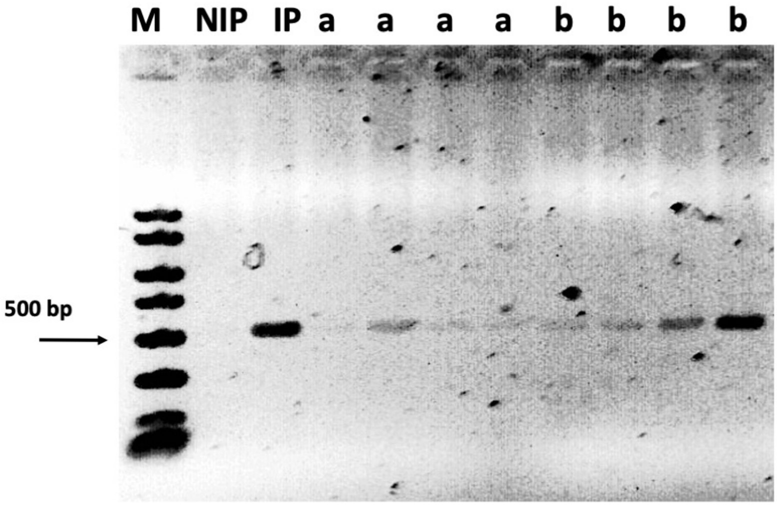

Figure S1. PCR amplification of *Verticillium dahliae* in tomato leaves. M: DNA molecular weight marker; NIP: non infected plants (Negative CTR); IP: infected plant non treated (Positive CTR); a: infected plant and treated with OLE-loaded CTNPs at concentration 1.41 mg/mL; b: infected plant and treated with OLE-loaded CTNPs at concentration 0.71 mg/mL.
